# Tumor-Associated Macrophages in Canine Oral and Cutaneous Melanomas and Melanocytomas: Phenotypic and Prognostic Assessment

**DOI:** 10.3389/fvets.2022.878949

**Published:** 2022-07-22

**Authors:** Ilaria Porcellato, Monica Sforna, Adriana Lo Giudice, Ilaria Bossi, Alice Musi, Alessia Tognoloni, Elisabetta Chiaradia, Luca Mechelli, Chiara Brachelente

**Affiliations:** ^1^Department of Veterinary Medicine, University of Perugia, Perugia, Italy; ^2^Faculty of Veterinary Medicine, University of Teramo, Teramo, Italy

**Keywords:** dogs, melanoma, tumor-associated macrophages, CD204, CD163, Iba1, MAC387, tumor-associated macrophages (TAMs)

## Abstract

The tumor microenvironment is a complex system, where neoplastic cells interact with immune and stromal cells. Tumor-associated macrophages (TAMs) are considered among the most numerically and biologically noteworthy cellular components in tumors and the attention on this cellular population has been growing during the last decade, both for its prognostic role and as a potential future therapeutic target. Melanoma, particularly the oral form, despite being one of the most immunogenic tumors, bears a poor prognosis in dogs and humans, due to its highly aggressive biological behavior and limited therapeutic options. The aims of this study are to characterize and quantify TAMs (using CD163, CD204, Iba1, and MAC387) in canine melanocytic tumors and to evaluate the association of these markers with diagnosis, histologic prognostic features, presence of metastases, and outcome, and to provide preliminary data for possible future therapies targeting TAMs. Seventy-two melanocytic tumors (27 oral melanomas, 25 cutaneous melanomas, 14 cutaneous melanocytomas, and 6 oral melanocytomas) were retrospectively selected and submitted to immunohistochemistry and double immunofluorescence. Double immunolabeling revealed that most CD163^+^ and CD204^+^cells co-expressed Iba1, which labeled also dendritic cells. Iba1 was instead rarely co-expressed with MAC387. Nevertheless, the expression of macrophagic markers showed a mild to moderate association among the four markers, except for CD204 and MAC387. The number of CD163^+^, CD204^+^, and MAC387^+^ cells was significantly higher in oral melanomas compared to oral melanocytomas (*p* < 0.001; *p* < 0.05 and *p* < 0.01, respectively), whereas Iba1 was differentially expressed in cutaneous melanomas and melanocytomas (*p* < 0.05). Moreover, CD163, IBA1 and MAC387 expression was associated with nuclear atypia and mitotic count. The number of CD163^+^cells was associated with the presence of metastases and tumor-related death in oral melanocytic tumors (*p* < 0.05 and *p* = 0.001, respectively).

## Introduction

Tumors are complex ecosystems composed of tumor cells, stromal cells, and immune cells; macrophages are recognized as one of the major components of these ecosystems (1–3). Still, in cancer, the role of this multifaceted cellular population, which serve as nexus between innate and adaptive immunity, is still poorly understood and studies addressing this topic in both human and veterinary medicine are surprisingly few. Actually, most of the oncology studies and large study groups are focused on lymphocytes and on their role in cancer immune response (4–10). Macrophages are a wide-ranging population of immune cells characterized by broad heterogeneity, high plasticity, and remarkable ability to sense changes in the surrounding microenvironment and respond to it. These cells are crucial in the detection, phagocytosis, and destruction of foreign and endogenous material, pathogens, but also cancer cells. Moreover, together with their phagocytic activity, they can participate in adaptive immunity by recruiting lymphocytes and presenting antigens to T cells (11–13).

Probably, the majority of TAMs are recruited macrophages (2), which are derived from circulating monocytes (14) and are in close contact with tumor cells, therefore the definition of tumor-associated macrophages (TAMs). Within the tumor microenvironment, these macrophages, together with myeloid-derived stem cells (MDSCs) are the major players in myeloid immunosuppression, which is believed to be triggered by cancer-related inflammation (15). TAMs can play different roles: promote angiogenesis and lymphangiogenesis, suppress antitumor immunity, particularly by suppressing cytotoxic T cell response, and stimulate cancer cells inducing proliferation, survival and invasion, and metastasis, therefore promoting tumor progression (3, 12).

The presence of TAMs in melanoma and their role in tumor progression and prognosis has been assessed in humans (16–20) and, in the last few years, the potential role of these cells as targets of future immunotherapies has been suggested (4, 21).

Recent studies have demonstrated that the role of TAMs depends on both their subtype (16, 17) and microanatomical localization. Besides, research has been focusing on the polarization of macrophages and also on their transcriptomic signature (2, 3, 14, 22).

In human cutaneous melanoma, high numbers of TAMs, especially when localized within the tumor cell nests, seem to enhance melanoma progression (18). Moreover, it has been shown that the production of midkine (MDK) can educate macrophages toward a tolerant phenotype, promoting CD8^+^cells dysfunction (20).

Studying TAMs both as prognostic indicators and as therapeutic targets in canine melanocytic tumors seems to be a promising path to follow to increase the current knowledge of their role in these tumors. Indeed, melanocytic tumors, despite being common in dogs, have sometimes unpredictable behavior and no curative available therapies (23–27); therefore, studies investigating additional valid prognostic features and feasible targets for new therapeutic strategies are fundamental.

Investigating TAMs in canine tumors is currently complicated by the limited availability of confirmed markers for canine macrophages to be used also on formalin-fixed and paraffin-embedded tissues.

Iba1 (also known as AIF-1) is a 17-kDa protein, which is specifically expressed by macrophages/microglia and upregulated during the activation of these cells (28). Some studies use Iba1 as a panmyeloid marker (29), whereas in other cases it is hypothesized that Iba1 identifies a M2-like phenotype of macrophages (30). In veterinary medicine, Iba1 has been recently used for the identification of TAMs in different canine tumors (31–33).

The scavenger receptor CD163 is expressed exclusively in macrophages and, less so, in monocytes; the expression of this protein is prompted by anti-inflammatory IL-6 and IL-10, whereas it is downregulated by pro-inflammatory stimuli (34). The expression of CD163 has a robust association with worse overall survival in human epithelial tumors and melanoma (35).

CD204 (also known as Scavenger Receptor-A, SR-A) is a transmembrane protein preferentially expressed on macrophages (36). The expression of this protein has shown a robust association with worse overall survival in epithelial neoplasms and melanoma in humans (35). Both CD163 and CD204 are recognized as possible M2-polarization markers.

Even though a rigorous categorization of macrophages is not possible, being them a continuous in terms of phenotype, a classification of these cells into M1-polarized macrophages (classically activated) and M2-polarized macrophages (anti-inflammatory) can be recognized. The process of polarization is the answer of macrophages to microenvironmental stimuli, by the acquisition of a specific phenotype. M1-polarized macrophages (pro-inflammatory) are critical in bacterial killing, tumor resistance, supporting tumor destruction and antigen presentation, and Th1 response (11). On the other hand, M2-polarized macrophages have anti-inflammatory properties, therefore favoring tumor growth and survival (35).

S100A8/A9 complex, also called calprotectin or MAC387, is expressed in phagocytic cells such as neutrophils, monocytes, dendritic cells, activated macrophages (but not non-activated macrophages), and platelets (37). This complex is intensely upregulated after trauma, in inflammatory processes, and stress (38). In a recent study, higher numbers of infiltrating MAC387-positive cells were found in metastasizing primary melanomas compared to non-metastasizing melanomas (39).

The aims of this study are:

- to characterize phenotypically and quantify TAMs within canine melanocytic tumors, using the currently available canine macrophagic markers IBA1, CD163, CD204, and MAC387 by immunohistochemistry and double immunofluorescence;
- to evaluate the association between the expression of different TAMs' markers with diagnosis, histologic prognostic features, presence of metastases, and outcome.

## Materials and Methods

### Case Selection

Seventy-two cases of canine melanocytic tumors were retrospectively selected from the archive of the Department of Veterinary Medicine of the University of Perugia.

Cases were included based on the following inclusion criteria:

- histological diagnosis of melanocytic tumor;
- in case of amelanotic tumor, diagnosis of melanocytic tumor confirmed by immunolabeling with Melan A and/or PNL2;
- available neoplastic tissue with an area >0.5 cm^2;^
- primary melanocytic tumor.

Cases were excluded if showing sampling or fixation artifacts.

A telephonic interview was conducted with the referring veterinarians to assess the presence of melanoma metastases and the cause of death. The minimum time from diagnosis to follow-up was set at 1 year. Dogs that were euthanized due to bad conditions caused by melanoma (inoperable local disease or metastases), were grouped together with dogs spontaneously dead because of the melanocytic tumor.

### Histopathological Examination

All the cases were re-examined by three pathologists (IP, MS, and CB) to confirm the original diagnosis following standard diagnostic criteria (40). Heavily pigmented tumors were bleached, following previously reported protocols (41).

The histological evaluation also included other features: mitotic count, nuclear atypia (40), pigmentation (expressed as a percentage of neoplastic pigmented cells), tumor thickness (42), and presence of melanophages. The first four features are considered significant in the prognosis of melanocytic neoplasms (40).

The presence of melanophages (macrophages containing a variable quantity of melanin pigment) was quantified as follows:

- 0: no melanophages observed in the examined sections;
- 1: small focal aggregates and/or scattered melanophages within the tumor (<5/HPF field);
- 2: multifocal aggregates of >5 melanophages, with areas of the tumor where no melanophages can be seen;
- 3: large multifocal to coalescing aggregates of melanophages.

### Western Blotting

Before immunohistochemical analysis, mouse anti-human Iba1 (Merck Millipore, Burlington, MA, US), anti-human CD163 (clone EDHu-1; Bio-Rad; Hercules, CA, US), and anti-human MRS-A/CD204 (clone SRA-E5;Cosmo Bio Co., LTD; Yuseong-Gu, Daejeon, Republic of Korea) antibodies were validated for detection of the canine proteins by Western blotting (WB), as previously reported (43). Forty mg of fresh tissues (lymph node harvested from a dog dead from trauma and placenta collected immediately after a spontaneous delivery) were homogenized in lysis buffer (Cell Signaling; Danvers, MA, US) containing protease inhibitor cocktail (Sigma-Aldrich) and centrifugated for 13,000 × g for 15 min at 4°C. The protein pellet was resuspended in phosphate-buffered saline (PBS) pH 7.4 and quantified using the Bradford assay. 50 μg of total proteins of each tissue were separated by polyacrylamide gel electrophoresis (SDS-PAGE) 12% T or 14% T and then transferred on nitrocellulose membranes. Specific protein bands were detected incubating membranes with the mouse anti-human CD163 (1:500), mouse anti-human CD204 antibody (1:300) or mouse anti-human Iba1 antibody (1:250) O.N. at 4°C and then at room temperature for 90 min with anti-mouse IgG polyclonal antibodies (1:5,000; Cell signaling). Immunoreactivity was evidenced by ECL system (Amersham ECL Prime Western Blotting Detection Reagent; Amersham, Amersham UK). The film images were acquired using a GS-800 imaging systems scanner (Bio-Rad).

### Immunohistochemistry

From formalin-fixed and paraffin-embedded samples, 5-μm sections were cut and mounted on poly-L-lysine-coated slides, which were then dewaxed and dehydrated. Immunohistochemistry was performed on serial sections with antibodies raised against CD163, CD204 (44), Iba1 (45), and MAC387 (33, 46, 47). In cases with more available histocassettes, the most representative was chosen, avoiding samples with large necrotic areas or ulceration. Positive controls were obtained from canine reactive lymph nodes for all the four antibodies used in this study; negative controls were run omitting the primary antibody and incubating control sections with TBS. Immunohistochemical protocols and antibodies used in this study are summarized in Table 1.

**Table 1:** Protocol details for immunohistochemistry.

**Antigen**	**Clone**	**Manufacturer**	**Antigen retrieval**	**Dilution**
CD163	EDHu-1	Bio-Rad	HIER; Tris-EDTA buffer; pH 9.0	1:100
CD204	SRA-E5	Cosmo Bio Co., LTD.	HIER; Tris-EDTA buffer; pH 9.0	1:100
Iba1	MABN92	Merck Millipore	HIER; Tris-EDTA buffer; pH 9.0	1:100
MAC387	M0747	Dako	HIER; Tris-EDTA buffer; pH 9.0	1:250

*HIER, heat-induced epitope retrieval*.

For each antibody, positive cells were manually counted in five consecutive HPF starting from hot spots, then the mean value was calculated; positive cells were considered only if they were intratumoral. Positive cells in the peripheral areas of the tumor or in the immediate peritumoral areas were not quantified but the presence of positive cells was recorded.

### Double Immunofluorescence

Double immunofluorescence was performed on five selected cases (two oral melanomas and three cutaneous melanomas). We selected Iba1 (as a panmyelocytic marker) and tested its co-expression with CD163, CD204, and MAC387. After dewaxing and dehydrating, the slides were incubated with a 3% H_2_O_2_ methanol solution. Antigen retrieval was performed as reported for immunohistochemistry and primary mouse antibodies (CD163, CD204 and MAC387) were incubated overnight at 4°C with the appropriate concentration. After TBS washing, a secondary goat anti-mouse antibody, conjugated with a red fluorochrome (Goat Anti-Mouse IgG H&L - Alexa Fluor® 647, Abcam) and with a 1:200 dilution, was applied for 2 h at room temperature. After a careful wash, a protein block step was performed prior to incubation with an anti-Iba1 antibody conjugated with a green fluorochrome (clone 20A12.1, Alexa Fluor® 488 Conjugate, Merck). Last, a drop of aqueous mounting medium containing DAPI (Abcam) was added and slides were incubated for 5 min at room temperature, before coverslip mounting.

Samples were evaluated using a fluorescent microscope Olympus BX51 equipped with the camera Nikon mod.DS-Qi2Mc. NIS-ELEMENTS D software was used for image acquisition and analysis.

### Statistical Analysis

Normality was assessed with a Shapiro–Wilk test for all continuous variables. Descriptive statistics were used to describe data; values are expressed as medians (Mdn) and interquartile range (IQR). Non-parametric tests were used to test hypotheses. The Kruskal-Wallis test and Mann–Whitney U test were performed to assess differences among groups. Correlation analysis was performed using Spearman's test (ρ). Descriptive statistics were performed using Microsoft Excel; other statistical tests were performed with IBM SPSS (version 21).

## Result

### Animals of the Study

The 72 melanocytic tumors retrospectively selected from our archive were obtained from 72 dogs. Twenty-seven cases were diagnosed as oral melanomas, 25 as cutaneous melanomas, 14 as cutaneous melanocytomas, and six as oral melanocytomas. Twenty-nine dogs were female, whereas 42 were male. In one case information on the sex of the animal was not available. The most represented breeds were mixed (25/72; 34.7%), German Shepherd (6/72; 8.3%), Labrador retriever (5/72; 6.9%), Golden retriever (4/72; 5.5%), English setter (3/72; 4.1%) and Yorkshire terrier (3/72; 4.1%).

The mean age of dogs at the moment of the diagnosis was 11 years (±3.1).

Of the 72 cases selected for the study, 55 had a complete follow-up. Nineteen cases were oral melanomas, 19 cutaneous melanomas, 12 cutaneous melanocytomas, and five oral melanocytomas.

Of the oral melanomas, 12 had distant metastases assessed at the moment of death, whereas five had a local relapse of the tumor after surgical excision. Two dogs did not have relapse nor metastases and one was still alive at the moment of the follow-up (>3 years), whereas the other dog died because of bronchomalacia a year later. Five out of six cases diagnosed with oral melanocytoma were still alive and only one case had a slowly growing relapse (>2 years after excision). In the group of cutaneous melanomas, six dogs died because of the tumor and, of these, five out of six had metastatic disease; only one case was euthanized due to the local invasion of a large mass infiltrating the temporo-mandibular region. Of the remaining 13 cases five died for other causes, whereas eight were still alive at the moment of the collection of follow-up data. Among cutaneous melanocytomas, one had metastasis to the local lymph node (diagnosed histologically), but after more than 3 years the dog is still alive and with no residual disease. In this case the authors agreed on the diagnosis of melanocytoma, based on histological features of the tumor and on what reported in human medicine, where nodal metastases can occur in up to 46% of cases of melanocytoma, despite benign behavior (48). Of the other 11 dogs with cutaneous melanocytomas, two died for other causes, whereas nine were still alive at the moment of the collection of follow-up data. Complete clinical information is reported in Supplementary Table 1.

### Histopathological Variables and Correlation With Outcome

Analysis of the histopathological variables showed a median mitotic count of 13.45 (IQR = 2.34–45.58). A statistical difference was detected among the four subgroups with different histological diagnosis (*p* < 0.001), in particular, oral melanomas (Mdn = 36.27; IQR = 11.7–60.84) statistically differ from oral melanocytomas (Mdn = 0.5; IQR = 0–1.46; *p* < 0.001) and cutaneous melanomas (Mdn = 23.4; IQR = 3.84–44.45) from cutaneous melanocytomas (Mdn = 2.17; IQR = 0–3; *p* < 0.001). Significant difference is lost among oral and cutaneous melanomas (*p* = 0.12) and oral and cutaneous melanocytomas (*p* = 0.13).

Considering all the cases of this study, the median value of pigmented cells was 30% (IQR = 5%−80%). Percentage of pigmented neoplastic cells in oral melanomas had a median value of 5% (IQR = 5%−30%), in cutaneous melanomas of 30% (IQR = 5%−70%), in oral melanocytomas of 92.50% (IQR = 82.50%−95.00%), and in cutaneous melanocytomas of 85% (IQR = 71.25%−100%). A statistically significant difference was observed among the four subgroups (*p* < 0.001); in particular, oral melanocytomas were significantly more pigmented than oral melanomas (*p* < 0.001) and the same was true for the cutaneous counterpart (*p* < 0.001).

Atypia in the present study group had a median value of 45% (IQR = 20%−80%), expressed as a percentage of cells showing signs of atypia on the total neoplastic cells. The median value of atypical cells in oral melanomas was 60% (IQR = 40%−80%) whereas in cutaneous melanomas was of 60% (IQR = 25%−85%). On the other hand, atypia in oral (Mdn = 7.50%; 4.50%–35.00%) and cutaneous melanocytomas (Mdn = 10%; IQR = 5%–26.25%) was lower. Oral melanomas showed a significantly higher percentage of atypical cells when compared to oral melanocytomas (*p* = 0.003) and the same was observed in cutaneous melanomas compared to cutaneous melanocytomas (*p* < 0.001).

Melanophages were graded from 0 to 3 and showed more frequently grade 3 or 2 in oral and cutaneous melanocytomas, whereas they were of a lower grade in the malignant counterpart. A statistical difference was observed in the four groups, with oral melanocytoma containing more melanophages than oral melanomas (*p* = 0.003) and cutaneous melanocytomas more melanophages than cutaneous melanomas (*p* = 0.005).

All these four histological variables were associated with outcome; in particular, higher mitotic counts and percentages of cellular atypia were associated with tumor-related death (*p* < 0.001 and *p* = 0.001, respectively). On the other hand, a higher percentage of pigmented cells and higher infiltration of melanophages were associated with a better outcome (*p* < 0.001 and *p* = 0.001, respectively). Considering only oral tumors (both melanomas and melanocytomas), atypia and pigmentation maintained their statistically significant association with tumor-related death (*p* < 0.05 for both variables), whereas the significance was lost for the number of melanophages (*p* = 0.26) and for the mitotic count (*p* = 0.07), although both parameters showed a trend. On the other hand, analyzing only cutaneous tumors, three of the four variables maintained their statistical significance (mitotic count: *p* < 0.005; pigmentation: *p* < 0.05; and melanophages: *p* = 0.005), whereas it was lost for atypia (*p* = 0.09). Histological data are reported in Supplementary Table 1.

### Validation of Antibodies Cross-Reactivity in Canine Tissues

**Figure 1:**
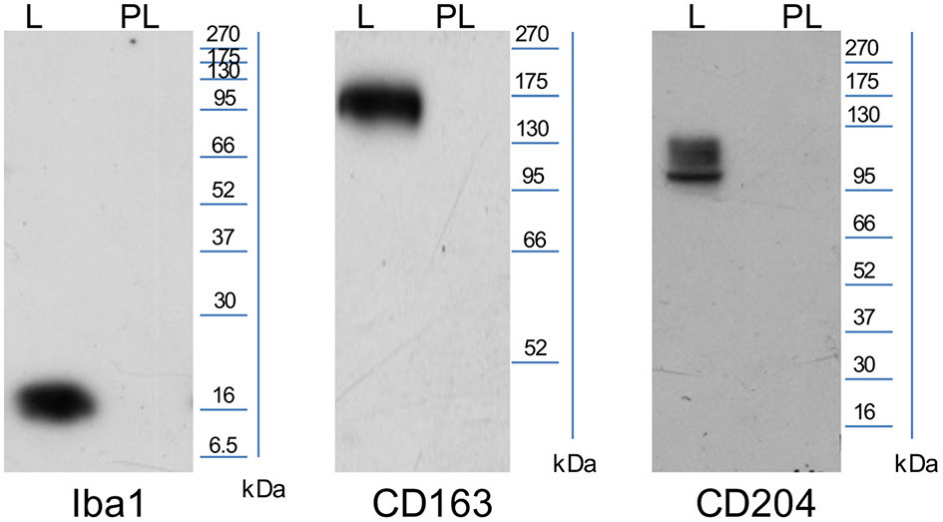
Representative images of WB performed using SDS-page 12% (CD163≃130–180 kDA, CD204≃90–120 kDA) and SDS-page 14% (Iba1≃16 kDa; L, lymph node and PL, placenta).

The WB images (Figure 1), obtained using anti-human Iba1 and anti-human CD163 antibodies, show a single band corresponding to the expected molecular weight 16 and 130–180 kDa, respectively, in the lymph node lane, whereas the anti-human CD204 antibody recognizes isoforms between 90 and 120 kDa. No bands were detected in the placenta lane for all antibodies tested.

### TAMs Markers Expression and Correlations With Diagnosis, Histopathological Variables, Metastases, and Outcome

#### Iba1 Expression Is Higher in Cutaneous Melanomas Compared to Cutaneous Melanocytomas

**Figure 2:**
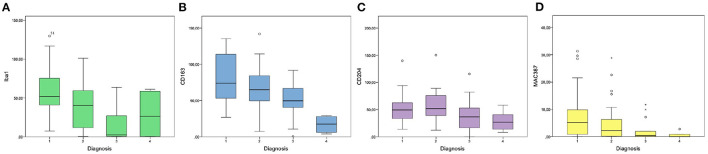
Graphics showing the results of intratumoral Iba1 **(A)**, CD163 **(B)**, CD204 **(C)**, and MAC387 **(D)** cell counts in the four groups of diagnosis (1: oral melanoma; 2: cutaneous melanoma; 3: cutaneous melanocytoma; 4: oral melanocytoma).

**Figure 3:**
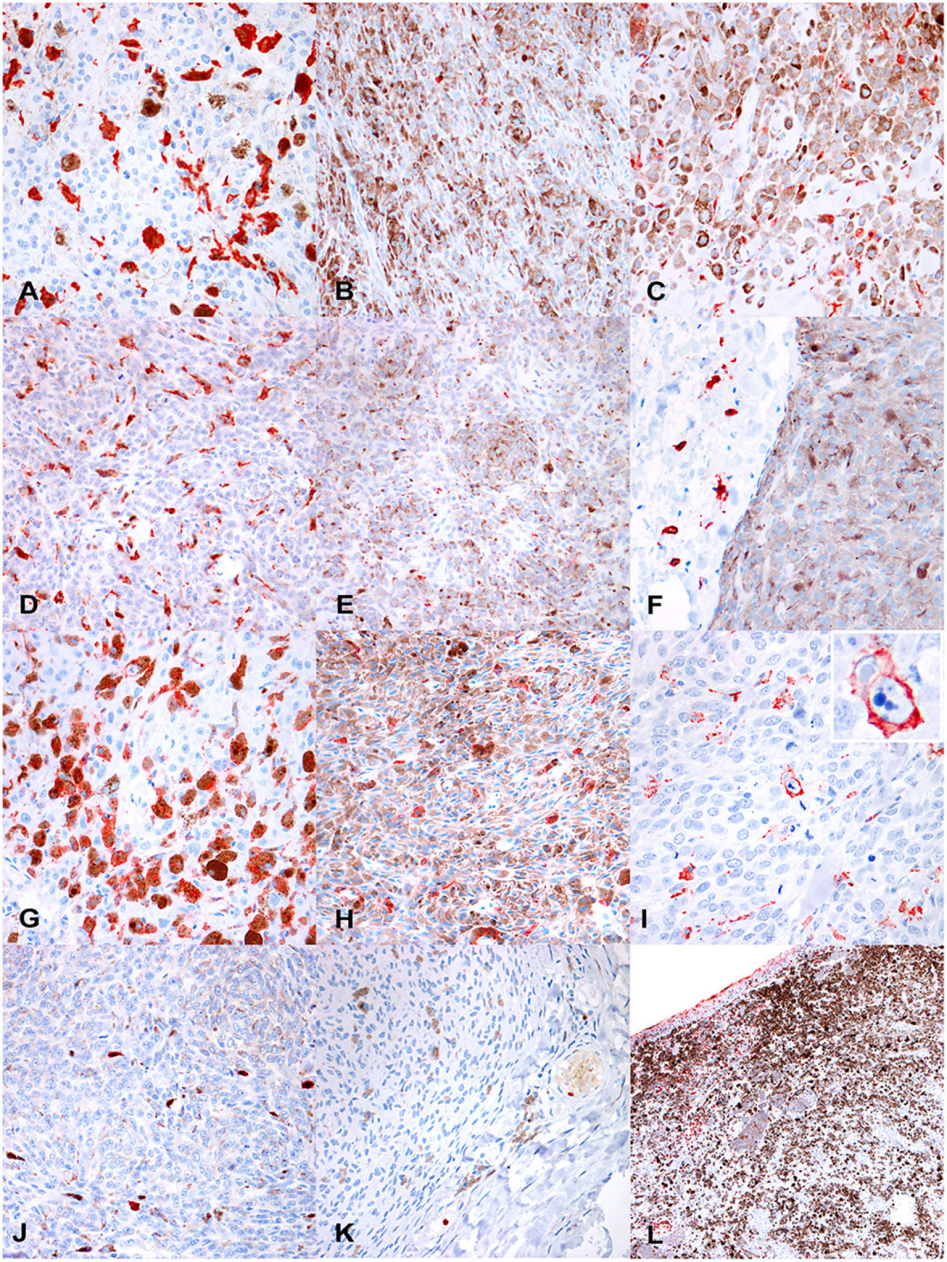
**(A)** Canine, cutaneous melanoma. Numerous intratumoral, large, Iba1^+^cells. Non neoplastic cells containing melanine (melanophages) are often faintly positive or negative (400 ×, AEC and hematoxylin). **(B)** Canine cutaneous melanocytoma. Rare intratumoral IBA1^+^ cells (400 ×, AEC and hematoxylin). **(C)** Canine cutaneous melanocytoma. In some tumors, Iba1^+^cells were smaller and with a more spindloid shape (400 ×, AEC and hematoxylin). Immunohistochemical results. **(D)** Canine, oral melanoma. Disseminated intratumoral CD163^+^cells, that show a polygonal-to-spindloid morphology (400 ×, AEC and hematoxylin). **(E)** Canine, cutaneous melanocytoma. Scattered CD163^+^ cells (400 ×, AEC and hematoxylin). **(F)** Canine, cutaneous melanocytoma. Extratumoral CD163^+^ cells, not infiltrating the tumor (400 ×, AEC and hematoxylin). **(G)** Canine, oral melanoma. Numerous, disseminated, intratumoral CD204^+^cells, that show a variable morphology, from rounded to elongated (400 ×, AEC and hematoxylin). **(H)** Canine, oral melanocytoma. Scattered CD204^+^cells among neoplastic cells. **(I)** Canine, cutaneous melanoma. Occasionally, CD204^+^cells, showed phagocytic activity (detail in the inset; 400 ×, AEC and hematoxylin). **(J)** Canine, oral melanoma. Occasional MAC387^+^cells, in deeper areas of the tumor far from areas of ulceration (400 ×, AEC and hematoxylin). **(K)** Canine, cutaneous melanocytoma. Occasional intravascular MAC387^+^cells, likely migrating into the tissue, whereas no MAC387^+^cells can be seen into the tumor (400 ×, AEC and hematoxylin). **(L)** Canine, oral melanoma. Numerous MAC387^+^cells (both macrophages and neutrophils) are present near superficial ulceration (200 ×, AEC and hematoxylin).

Iba1 was expressed in the cytoplasm of macrophages and monocytes. Cells with prominent cytoplasmic projections were interpreted as dendritic cells. Also, Iba1 labeling was variable in melanophages, with the majority of these cells being weakly or not labeled. The median number of Iba1^+^cells/HPF was 42.8 (IQR = 29.3–61.31). Kruskal-Wallis test indicated a statistically significant difference among the four diagnosis groups (*p* = 0.001; Figure 2A). Iba1^+^cells/HPF in oral melanomas (Mdn = 51.8; IQR = 40.9–75.3) did not differ statistically from oral melanocytomas (Mdn = 26.3; IQR = 3.05–54.3; *p* = 0.76); on the contrary, a statistical difference was observed between cutaneous melanomas (Mdn = 40.4; IQR = 11.6–59.4; Figure 3A) and melanocytomas (Mdn = 2.4; IQR = 0.65–22.5; Figures 3B,C; *p* < 0.05).

#### CD163 Expression Is Higher in Oral Melanomas Compared to Oral Melanocytomas

Immunolabeling for CD163 was observed as strong membranous staining. Cells were morphologically defined as macrophages or monocytes, according to the size of the cell, the amount of cytoplasm, and size and location of the nucleus. Melanophages were variably positive to CD163, showing frequently a weaker immunolabeling compared to smaller monocytes. CD163^+^macrophages showed a variable morphology, from dendritic, to polygonal or rounded, to spindloid; also, the dimension of positive cells was highly variable, being larger when cells were filled with pigment or vacuoles. CD163^+^cells were frequently scattered as single elements throughout the tumor; aggregates were instead more frequently observed near areas of necrosis.

The median number of CD163^+^cells/HPF was 64.25 (IQR = 42.5–84.35) and showed a statistically significant difference among the four diagnosis groups (oral melanoma, cutaneous melanoma, cutaneous melanocytoma and oral melanocytoma; *p* < 0.001; Figure 2B). Oral melanomas were characterized by the highest number of CD163^+^cells/HPF (Mdn = 73.9; IQR = 54.5–108.9; Figure 3D), followed by cutaneous melanomas (Mdn = 64.9; IQR = 51.7–83.36), cutaneous melanocytomas (Mdn = 49.6; IQR = 41.1–64.35), and oral melanocytomas (Figure 3E; Mdn = 17.4; IQR = 8.1–25.95). Statistical significance increased by running a Mann–Whitney *U*-test comparing oral melanomas to oral melanocytomas (*p* < 0.001), whereas it was lost comparing cutaneous melanomas to cutaneous melanocytomas (*p* = 0.77). Four cases were not examined due to the lack of tissue after serial recuts. In some cases, the presence of extratumoral CD163^+^cells was observed, in face of a non-infiltrated tumor (Figure 3F).

#### CD204 Expression Is Higher in Oral Melanomas Compared to Oral Melanocytomas

CD204^+^ cells showed a marked membranous positivity; the immunolabeled elements were cytologically interpreted as macrophages and monocytes, with variable shapes and dimensions. Similarly to CD163, melanophages were often characterized by a faint positivity.

The median number of CD204^+^cells within the tumors was 41.6 cells/HPF (IQR = 30–63.8). Kruskal–Wallis test did not reveal a statistically significant difference among the four groups, but a trend was identified (*p* = 0.058; Figure 2C). Therefore, a Mann–Whitney *U*-test was run comparing oral melanomas to oral melanocytomas and cutaneous melanomas to cutaneous melanocytomas. Oral melanomas showed a statistically significant higher number of CD204^+^cells/HPF (Mdn = 49.2; IQR = 33.5–62.6; Figure 3G), when compared to their benign counterpart (Mdn = 26.7; IQR = 16.4–37.75; Figure 3H; *p* < 0.04). On the contrary, the difference was not statistically significant between cutaneous melanomas and melanocytomas (*p* = 0.77; Mdn = 51.8; IQR = 39.3–74.45 and Mdn = 36.4; IQR = 18.45 and 50.2, respectively). Interestingly, CD204 immunoreactivity in cells showing evidence of phagocytosis was frequently observed within malignant tumors (Figure 3I).

#### MAC387 Expression Is Higher in Oral Melanomas Compared to Oral Melanocytomas

MAC387 expression was observed as a strong, diffuse cytoplasmic immunolabeling in both mononucleated cells (interpreted as macrophages) and polymorphonucleated cells, mostly neutrophils. Monocytes were observed mostly in deeper areas of the tumor and were few (Figure 3J). Melanophages were invariably negative for MAC387; whereas intravascular neutrophils and monocytes showed strong immunolabeling (Figure 3K). Superficial areas with ulceration were usually heavily infiltrated by neutrophils (Figure 3L).

The mean number of MAC387 was 2.06 cells/HPF and was significantly different among the four diagnosis groups (*p* < 0.05; Figure 2D). In particular, oral melanomas marker expression (Mdn = 5.17; IQR = 0.85–9.78) was significantly higher than in oral melanocytomas (Mdn = 0; IQR = 0–0.6; *p* < 0.01). Instead, a significance was not reached between cutaneous melanomas (Mdn = 2.19; IQR = 0.21–6.27) and melanocytomas (Mdn = 0.4; IQR = 0–1.91; *p* = 0.76).

### Correlations Between TAMs Markers Expression and Histopathological Variables

CD163 expression showed a moderate positive correlation with the expression of CD204 and Iba1 (*p* < 0.001; ρ = 0.472 and ρ = 0.521, respectively). The correlation with MAC387 was instead weak (*p* < 0.01; ρ = 0.329). A moderate positive correlation was observed also with prognostic histologic markers of nuclear atypia (*p* < 0.001; ρ = 0.416) and mitotic count (*p* < 0.001; ρ = 0.446). A weak negative correlation was instead observed with pigmentation (*p* < 0.01; ρ = −0.368) and with the presence of melanophages (*p* < 0.05; ρ = −0.265).

The expression of CD204, instead, showed a positive weak-to-moderate correlation with Iba1 expression (*p* = 0.001; ρ = 0.391); a weak expression was observed also with nuclear atypia and mitotic count (*p* < 0.05; ρ = 0.251 and ρ = 0.276, respectively).

Iba1 expression was moderately correlated with nuclear atypia (*p* < 0.001; ρ = 0.403) and had a weak-to-moderate correlation with the mitotic count (*p* = 0.001; ρ = 0.373). A moderate negative correlation was seen with pigmentation and melanophages (*p* < 0.001; ρ = −0.481 and ρ = −0.410, respectively).

MAC387 showed a moderate positive correlation with mitotic count (*p* < 0.001; ρ = 0.428) and a weak correlation with nuclear atypia (*p* < 0.01; ρ = 0.310). A weak negative correlation was seen with pigmentation and melanophages (*p* = 0.001 and *p* < 0.05; ρ = −0.368 and ρ = −0.288, respectively).

The results of the correlation analysis are reported in Table 2.

**Table 2:** Correlation analysis (Spearman rank correlation coefficient, ρ).

	**CD204**	**Iba1**	**MAC387**	**Nuclear atypia**	**Mitotic count**	**Pigmentation**	**Melanophages**
CD163	0.472^**^	0.521^**^	0.329^**^	0.416^**^	0.446^**^	−0.368^**^	−0.265^*^
CD204	1.000	0.391^**^	0.184	0.251^*^	0.276^*^	−0.209	−0.069
Iba1		1.000	0.268^*^	0.403^**^	0.373^**^	−0.481^**^	−0.410^**^
MAC387			1.000	0.310^**^	0.428^**^	−0.368^**^	−0.288^*^
Nuclear atypia				1.000	0.597^**^	−0.319^**^	−0.299^*^
Mitotic count					1.000	−0.671^**^	−0.611^**^
Pigmentation						1.000	0.831^**^

* *p < 0.05*.

** *p < 0.01*.

The correlation with tumor thickness was assessed only for cutaneous tumors. No correlation was observed with CD163 and CD204, whereas a moderate positive correlation was present with Iba1 and MAC387 (*p* < 0.05; ρ = 0.450 and 0 ρ = 0.451, respectively).

### Correlations Between TAMs Markers Expression and Metastases and Outcome

Considered altogether, Iba1, CD163, and MAC387 expression was significantly associated with tumor-related death associated with death due to melanoma (*p* < 0.01 for Iba1 and *p* < 0.05 for CD163 and MAC387). Furthermore, Iba1 expression was associated with the presence of metastases (*p* < 0.05), and, although not statistically significant, CD163 expression showed a trend (*p* = 0.73).

When we performed the analysis on oral tumors and cutaneous tumors separately, CD163 confirmed its association with metastases and with death for melanoma in the oral group (*p* < 0.05 and *p* = 0.001, respectively). On the other hand, none of the markers tested was associated with metastases or death for melanoma in the group of cutaneous tumors. A mild trend was observed for CD204 (*p* = 0.067).

### TAMs Markers Co-Expression

**Figure 4:**
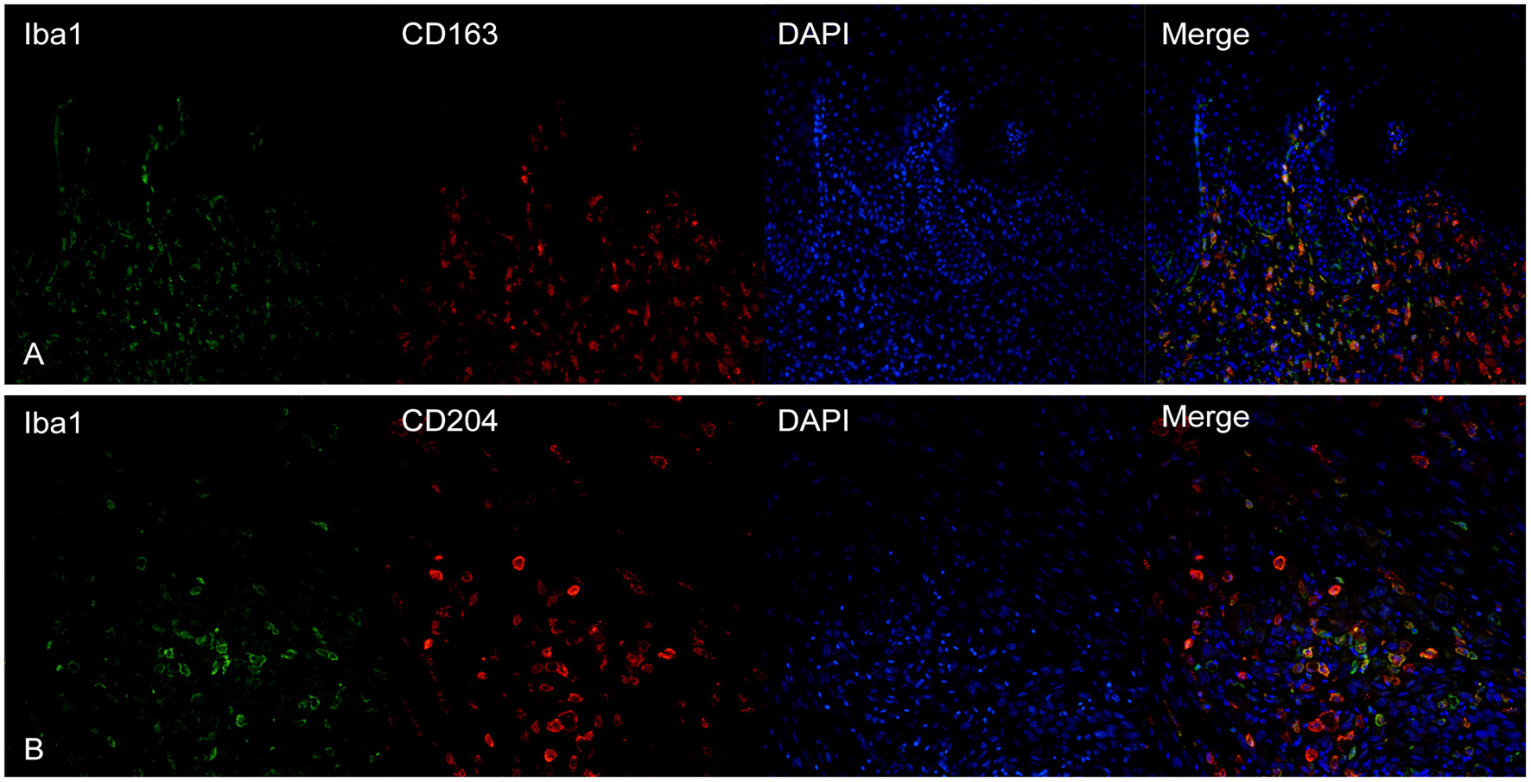
**(A)** Canine, oral melanoma. Colocalization of Iba1 (green) and CD163 (red) shows a partial co-expression of the two markers. Intraepithelial dendritic cells are negative for CD163 but positive for Iba1. **(B)** Canine, oral melanoma. A partial overlap of Iba1 (green) and CD204 (red) was observed. Nuclear counterstain was performed with DAPI.

CD163 showed a frequent co-expression with Iba1, but the two cellular populations did not overlap completely (Figure 4A). Large polygonal cells with abundant cytoplasm, interpreted as macrophages, often showed a marked positivity for CD163 but were negative for Iba1. Instead, cells with dendritic morphology, as well as intraepithelial dendritic cells, were positive for Iba1 but negative for CD163.

CD204 and Iba1 showed a co-expression profile similar to CD163-Iba1 and, also in this case, cells with a macrophagic morphology, were usually positive for CD204 but not for Iba1 (Figure 4B). Intraepithelial dendritic cells were variably positive, being instead strongly positive for Iba1.

**Figure 5:**
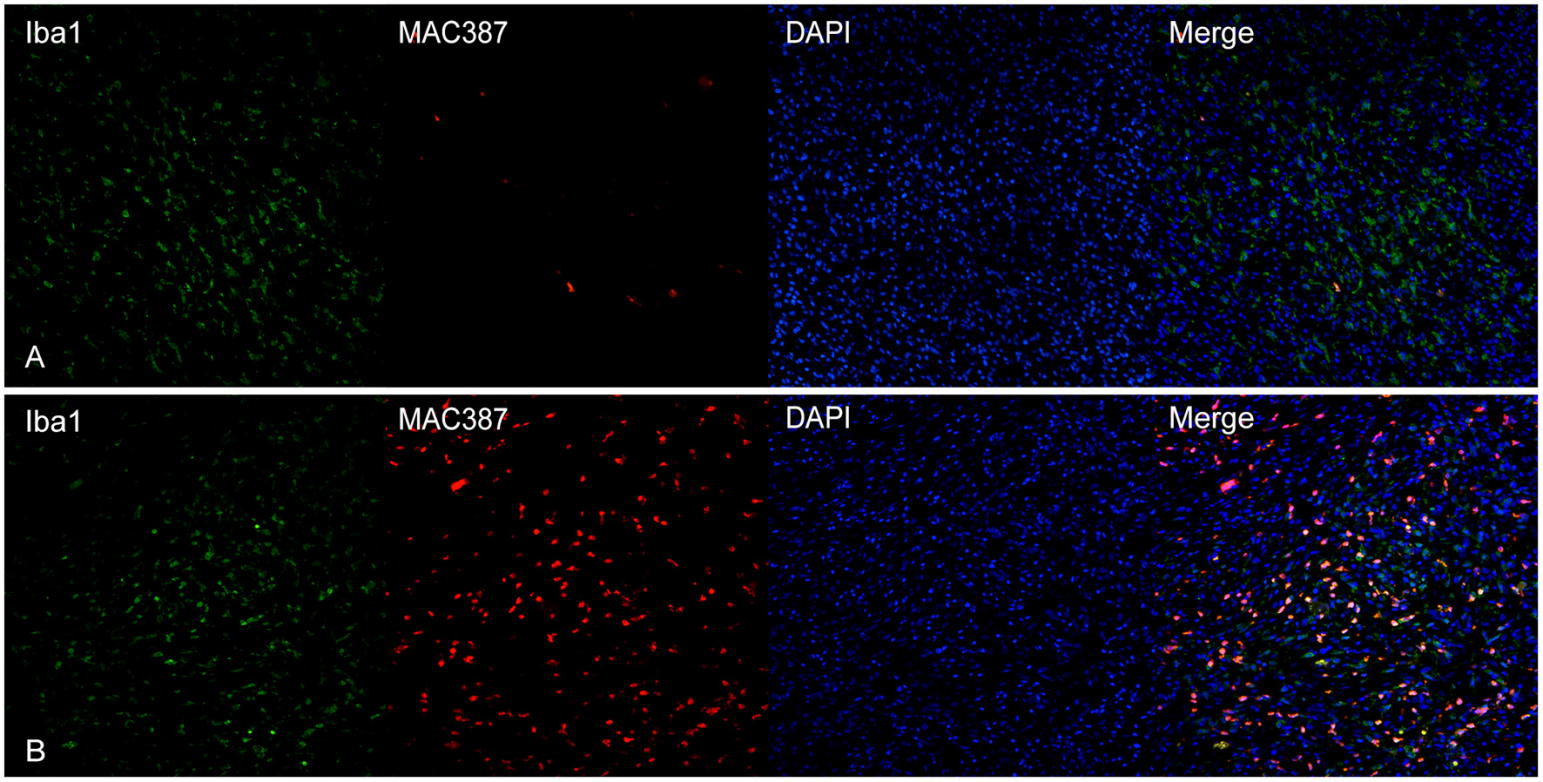
**(A)** Canine, oral melanoma. Colocalization of Iba1 (green) and MAC387 (red). In melanomas, MAC387^+^cells were few and often did express also Iba1. **(B)** Canine, cutaneous melanoma. In areas near superficial ulceration, the number of cells co-expressing Iba1 and MAC387 was elevated. Nuclear counterstain was performed with DAPI.

MAC387 showed a variable co-expression with Iba1; usually, scant double-labeled Iba1-MAC387^+^cells were seen within the tumor (Figure 5A). On the other hand, near superficial areas of ulceration, double-positive cells increased (Figure 5B).

## Discussion

The tumor microenvironment is a rich and complex system, where neoplastic cells interact with immune cells and stroma; TAMs are considered among its most numerically and biologically significant cellular components (1, 2).

The present study focuses for the first time on TAMs in canine melanocytic tumors using different macrophagic markers, namely Iba1, CD163, CD204, and MAC387. The correlation of these markers' expression with prognostic histologic features, presence of metastases, and outcome are also assessed.

Overall, we observed a moderate association between CD163 and CD204, CD163 and Iba1 and a weak-to-moderate association between CD204 and Iba1. These results seem to indicate an association and at least a partial overlap of the expression of these markers, as confirmed by the co-expression analysis through double immunofluorescence. MAC387 has been considered a reliable macrophagic marker for years in veterinary medicine (49, 50); however, our present results show that this protein is expressed only on a small percentage of intratumoral macrophages and that therefore it should not be considered a sensitive marker for this cellular population. The expression of this marker is likely limited to recently recruited macrophages, as reported in other studies (51). Double labeling showed an increase of the presence of a population of cells co-expressing MAC387 and Iba1 near areas of active inflammation (near superficial ulcers), together with neutrophilic infiltration. This finding may indicate a possible M1-polarization of macrophages expressing MAC387, and support the hypothesis of a recent migration of these cells to the affected tissue (51, 52).

In the present study, the number of CD163^+^cells/HPF was significantly higher in oral melanomas when compared to oral melanocytomas; moreover, a moderate positive correlation was observed with nuclear atypia and mitotic index, together with a negative correlation with pigmentation and the presence of melanophages. Taken together, these results seem to indicate an increase of CD163^+^cells in association with malignant prognostic markers (nuclear atypia and mitotic index), and an inverse correlation with pigmentation, usually associated with a better outcome (53).

CD163 expression was also higher in melanomas with evidence of metastasis and in tumors from dogs that died because of the tumor. These results are similar to human melanoma, where CD163 expression has shown a robust association with worse overall survival (18, 35). An increase in CD163 expression is similarly reported in other high-grade canine tumors, such as lymphomas and gliomas (33, 46). CD163 is considered a marker for M2-polarized macrophages, hence, the presence of numerous CD163^+^cells supports the hypothesis of an activation of immunosuppression and immunoescape mechanisms within the tumor microenvironment also in canine melanomas, as we previously hypothesized by testing the expression of IDO, Foxp3, and CTLA-4 (41, 54).

CD204 was significantly more expressed in oral melanomas when compared to the benign counterpart, but this difference was not observed in the cutaneous melanocytic tumors; overall, a mild correlation with nuclear atypia and mitotic index was present. These results seem to indicate that also this marker may be associated with prognosis, as reported in other studies in both humans and dogs (17, 31, 33, 35), but further studies with a larger cohort with a complete follow-up are needed to substantiate these results in canine melanomas.

Iba1 expression showed a significant difference between cutaneous melanomas and melanocytomas, but not in the oral counterpart and was moderately correlated with nuclear atypia. This marker is considered a pan-myeloid in humans and has shown a negative impact on survival in patients with glioblastoma, despite not reaching a statistical significance (29); moreover, Iba1 has been suggested as a prognostic marker and a possible therapeutic target in human hepatocellular carcinomas (30). In veterinary medicine, a linear correlation between Iba1 expression and the mitotic count has been observed in canine soft tissue sarcomas (32). As a matter of fact, the role of Iba1^+^cells in tumor microenvironment has not been completely elucidated, but its favoring function in a pro-tumor immune microenvironment and as a negative prognostic factor seems likely.

Last, MAC387 was more expressed in oral melanomas compared to melanocytomas and was moderately associated with the mitotic count. This finding may be associated with the presence of ulceration, which was more common in oral melanomas compared to the benign counterpart, where numerous cells co-expressing MAC387 and Iba1 were observed by means of double immunofluorescence. Despite performing cell counts far from areas of ulceration, the presence of these cells may be justified by these events, even if ulceration is not immediately adjacent. Anyway, it is important to stress that, in general, the number of MAC387^+^cells was noticeably lower when compared to the other markers. The exact role of MAC387^+^ macrophages is still not clear; in some studies, they are reported as M1-polarized macrophages (33, 55), whereas some other results seem to address them as an M2-like population (39).

The lack of statistical significance evidenced between cutaneous melanomas and cutaneous melanocytomas for CD163, CD204 and MAC387 expression, may reflect the unpredictable behavior of the first group of tumors. Also, it can be hypothesized that some tumors, histologically classified as cutaneous melanomas, should be reclassified as melanocytomas, also based on the biological behavior. Hence, a review of the histological prognostic features (40) for cutaneous melanocytic tumors might be considered, in order to increase their accuracy.

The partial co-expression of TAM markers evidenced by our study also confirms the complexity of TAMs within canine melanocytic tumors, particularly within the malignant ones. Therefore, further investigation is recommended to better identify the roles of each macrophagic subpopulation and to assess their use as prognostic markers. M2 markers are usually associated with worse overall survival in human neoplastic diseases, but also the anatomical region may influence these associations (27). This may partially explain the differences we observed between oral and cutaneous tumors and deserves further investigation.

The present study underlines the potential prognostic role of TAMs in canine melanocytic tumors.

The main limits that we encountered are due to the lack of specific macrophagic markers in dogs; for instance, CD68, which is considered a pan-macrophagic marker in humans, does not seem suitable for canine FFPE samples. To understand and define the universe of TAMs, it is necessary to complement these morphological studies with biomolecular approaches and our group is currently working on the phenotypic and proteomic characterization of macrophages and histiocytes in canine melanocyte tumors.

Another problem is associated with the growing difficulty in having homogeneous groups to perform survival analysis. Regarding this, some of our groups were not numerically homogeneous (i.e. oral melanocytomas). However, this reflects a real epidemiological difference and confirms the need to collect more data on these rare categories of melanocytic tumors. Different therapeutic approaches can deeply alter the results of survival analyses; therefore, we decided to analyze the prognostic significance of TAMs markers only in association with the presence of metastasis and on the outcome.

The results of this study confirm that TAMs may represent an interesting target for future immunotherapeutic approaches in dogs; moreover, these data may be useful for comparative studies, particularly with human oral melanoma, for which the canine counterpart is recognized as a valuable spontaneous model (24, 56–59). CD163 expression has shown a significantly different profile and potential prognostic significance in oral melanomas and melanocytomas, suggesting an immunosuppressive and pro-tumorigenic role of this cellular subpopulation. Moreover, the functional characterization of CD163^+^ and CD204^+^ macrophages in oral melanocytic tumors and of Iba1^+^ cells in cutaneous tumors may help to deepen our knowledge of the immune pathways involved in melanoma progression and mediated by TAMs. Overall, this study shows that TAMs in canine melanocytic tumors deserve further investigations, not only as prognostic biomarkers, but also as future feasible therapeutic targets for both canine and human disease.

## Data Availability Statement

The raw data supporting the conclusions of this article will be made available by the authors, without undue reservation.

## Ethics Statement

Ethical review and approval was not required for the animal study because the study was performed on archive FFPE material harvested for diagnostic or curative scopes. Written informed consent was obtained from the owners for the participation of their animals in this study.

## Author Contributions

IP, MS, and CB conceived the presented idea, classified, and selected the tumor cases. IP wrote the manuscript. LM supervised the findings of this work. AG, IB, and AM performed the experiments on tumor tissues. EC and AT validated the antibodies by western blotting. IP, MS, IB, and AM performed cell counting. IP performed the statistical analysis. All authors discussed the results and contributed to the final manuscript.

## Conflict of Interest

The authors declare that the research was conducted in the absence of any commercial or financial relationships that could be construed as a potential conflict of interest.

## Publisher's Note

All claims expressed in this article are solely those of the authors and do not necessarily represent those of their affiliated organizations, or those of the publisher, the editors and the reviewers. Any product that may be evaluated in this article, or claim that may be made by its manufacturer, is not guaranteed or endorsed by the publisher.
