# Supplementary material for: Tumor-Associated Macrophages in Canine Oral and Cutaneous Melanomas and Melanocytomas: Phenotypic and Prognostic Assessment
Source: Front Vet Sci. 2022 Jul 22;9:878949. doi: 10.3389/fvets.2022.878949 (PMC9355725; doi:10.3389/fvets.2022.878949)
Supplement: Supplementary file 1 [file Data_Sheet_1.docx]

|  | **CLINICAL INFORMATION** | | | | | **HISTOLOGICAL DATA** | | | | |
| --- | --- | --- | --- | --- | --- | --- | --- | --- | --- | --- |
| **Case** | **Breed** | **Age** | **Sex** | **Site of the primary lesion** | **Mets** | **Histological diagnosis** | **MC** | **ATY** | **PG** | **MF** |
| 1 | Mixed breed | 9 | F | perianal | n/a | Cutaneous melanoma | 29,25 | 40 | 5 | 1 |
| 2 | Mixed breed | 15 | F | face (cheek) | no | Cutaneous melanoma | 49,14 | 90 | 20 | 2 |
| 3 | Pekingese | 10 | M | gingiva | n/a | Oral melanoma | 86,58 | 50 | 0 | 0 |
| 4 | Poodle | 14 | M | gingiva | L | Oral melanoma | 26,91 | 40 | 0 | 1 |
| 5 | Dachshund | 10 | M | gingiva | n/a | Oral melanoma | 36,27 | 90 | 80 | 3 |
| 6 | Mixed breed | n/a | M | gingiva | no | Oral melanocytoma | 1 | 3 | 90 | 2 |
| 7 | Mixed breed | 14 | F | lip | n/a | Oral melanoma | 40,95 | 70 | 40 | 2 |
| 8 | Mixed breed | 12 | M | pawpad | D | Cutaneous melanoma | 23,4 | 90 | 5 | 1 |
| 9 | German shepherd | 9 | M | digit | no | Cutaneous melanoma | 2 | 60 | 80 | 3 |
| 10 | German shepherd | 1 | F | palpebra | no | Cutaneous melanoma | 15,21 | 30 | 70 | 1 |
| 11 | Labrador retriever | 14 | M | forelimb | n/a | Cutaneous melanoma | 2,34 | 100 | 90 | 2 |
| 12 | Mixed breed | 11 | M | hindlimb | no | Cutaneous melanoma | 0 | 10 | 100 | 3 |
| 13 | Mixed breed | 10 | n/a | forelimb | no | Cutaneous melanoma | 0 | 20 | 80 | 2 |
| 14 | n/a | 9 | F | cheek | no | Cutaneous melanocytoma | 1,17 | 10 | 80 | 3 |
| 15 | Mixed breed | 9 | M | lip (skin) | no | Cutaneous melanocytoma | 2,34 | 5 | 100 | 3 |
| 16 | Mixed breed | 12 | M | perineum | no | Cutaneous melanocytoma | 2,34 | 30 | 100 | 2 |
| 17 | Labrador retriever | 9 | M | skin n/a | n/a | Cutaneous melanoma | 4,68 | 30 | 5 | 0 |
| 18 | English setter | 7 | M | gingiva | n/a | Oral melanoma | 11,7 | 20 | 2 | 1 |
| 19 | Mixed breed | 15 | F | gingiva | D | Oral melanoma | 8,19 | 30 | 90 | 3 |
| 20 | Mixed breed | 15 | M | lip | L | Oral melanoma | 54,99 | 90 | 0 | 0 |
| 21 | n/a | n/a | F | gingiva | n/a | Oral melanoma | 134,55 | 80 | 5 | 1 |
| 22 | Beagle | 13 | M | buccal mucosa | no | Oral melanoma | 46,8 | 100 | 35 | 2 |
| 23 | Gordon setter | 12 | M | shoulder | L | Cutaneous melanoma | 21,06 | 60 | 15 | 1 |
| 24 | Boxer | 14 | F | abdomen | D | Cutaneous melanoma | 140,4 | 90 | 30 | 1 |
| 25 | English setter | 11 | M | scrotum | no | Cutaneous melanocytoma | 2 | 30 | 80 | 2 |
| 26 | Bernese mountain dog | 6 | M | buccal mucosa | D | Oral melanoma | 19,89 | 50 | 20 | 2 |
| 27 | Shih tzu | n/a | M | buccal mucosa | L | Oral melanoma | 169,65 | 80 | 0 | 0 |
| 28 | Mixed breed | n/a | F | neck | no | Cutaneous melanoma | 56,12 | 80 | 50 | 2 |
| 29 | Springer spaniel | n/a | F | abdomen | no | Cutaneous melanoma | 9,36 | 95 | 5 | 1 |
| 30 | Shih tzu | 14 | M | gingiva | no | Oral melanoma | 46,8 | 90 | 20 | 1 |
| 31 | n/a | 12 | F | perianal | D | Cutaneous melanoma | 39,76 | 30 | 10 | 1 |
| 32 | Golden retriever | n/p | M | tongue | no | Oral melanoma | 53,82 | 55 | 0 | 1 |
| 33 | German shepherd | n/a | M | interdigital | no | Cutaneous melanocytoma | 3,51 | 60 | 100 | 3 |
| 34 | Golden retriever | n/a | F | gingiva | D | Oral melanoma | 60,84 | 60 | 2 | 1 |
| 35 | German shepherd | 10 | M | nose | no | Cutaneous melanocytoma | 2,34 | 20 | 60 | 2 |
| 36 | Golden retriever | 7 | F | sublingual | no | Oral melanoma | 79,56 | 80 | 0 | 0 |
| 37 | Golden retriever | 9 | M | lip | no | Oral melanoma | 31 | 7 | 1 | 1 |
| 38 | Miniature pinscher | 9 | F | perianal | no | Cutaneous melanocytoma | 0 | 5 | 50 | 3 |
| 39 | West highland white terrier | n/a | M | buccal mucosa | L | Oral melanoma | 33,93 | 90 | 40 | 0 |
| 40 | Mixed breed | 12 | M | lip | no | Cutaneous melanoma | 9,36 | 50 | 65 | 3 |
| 41 | Labrador retriever | 15 | M | digit | n/a | Cutaneous melanoma | 72 | 70 | 0 | 1 |
| 42 | Yorkshire terrier | 18 | F | forehead | D | Cutaneous melanoma | 76,05 | 20 | 5 | 1 |
| 43 | Yorkshire terrier | 13 | F | buccal mucosa | no | Oral melanoma | 31,59 | 90 | 5 | 1 |
| 44 | Mixed breed | 10 | F | buccal mucosa | D | Oral melanoma | 7,02 | 20 | 0 | 1 |
| 45 | English setter | 3 | F | buccal mucosa | D | Oral melanoma | 11,7 | 30 | 35 | 2 |
| 46 | Cocker spaniel | 15 | F | lip | no | Oral melanocytoma | 1,17 | 80 | 60 | 2 |
| 47 | Mixed breed | 9 | M | abdomen | no | Cutaneous melanoma | 24,57 | 90 | 70 | 2 |
| 48 | Mixed breed | 12 | F | lip | no | Oral melanoma | 122,85 | 30 | 0 | 1 |
| 49 | Mixed breed | 14 | F | lip (skin) | no | Cutaneous melanoma | 170,82 | 60 | 30 | 1 |
| 50 | Mixed breed | 8 | F | buccal mucosa | L | Oral melanoma | 5,85 | 60 | 5 | 1 |
| 51 | Mixed breed | 11 | F | buccal mucosa | no | Oral melanoma | 6,5 | 80 | 10 | 1 |
| 52 | Labrador retriever | 7 | M | interdigital | n/a | Cutaneous melanocytoma | 0 | 3 | 100 | 2 |
| 53 | Mixed breed | 8 | M | stifle | no | Cutaneous melanoma | 39 | 5 | 0 | 0 |
| 54 | Flat coated retriever | 11 | M | gingiva | L | Oral melanoma | 44,46 | 70 | 5 | 1 |
| 55 | Miniature pinscher | 12 | F | lip (skin) | no | Cutaneous melanocytoma | 1,17 | 5 | 90 | 2 |
| 56 | Pug | 11 | M | skin n/a | no | Cutaneous melanocytoma | 0 | 5 | 95 | 3 |
| 57 | n/a | n/a | F | digit | no | Cutaneous melanoma | 37,44 | 80 | 60 | 2 |
| 58 | Dobermann | n/a | M | lip | no | Oral melanocytoma | 2,34 | 5 | 95 | 2 |
| 59 | Mixed breed | 10 | M | interdigital | L | Cutaneous melanocytoma | 3 | 5 | 95 | 3 |
| 60 | Mixed breed | 6 | F | thigh | no | Cutaneous melanocytoma | 5,85 | 10 | 5 | 1 |
| 61 | Mixed breed | n/a | F | head | no | Cutaneous melanoma | 30,42 | 60 | 0 | 0 |
| 62 | American Staffordshire Terrier | 10 | M | nose | n/a | Cutaneous melanoma | 3 | 7 | 0 | 0 |
| 63 | Labrador retriever | 10 | M | interdigital | no | Cutaneous melanoma | 24,57 | 40 | 55 | 3 |
| 64 | Mixed breed | 15 | M | buccal mucosa | no | Oral melanoma | 85 | 80 | 10 | 0 |
| 65 | Cocker spaniel | 15 | F | buccal mucosa | no | Oral melanoma | 15,21 | 40 | 5 | 1 |
| 66 | Rottweiler | 10 | F | hard palate | no | Oral melanocytoma | 0 | 20 | 90 | 3 |
| 67 | Dachshund | 14 | M | palate | no | Oral melanocytoma | 0 | 5 | 95 | 3 |
| 68 | Chow chow | 9 | M | glottis | no | Oral melanocytoma | 0 | 10 | 95 | 2 |
| 69 | German shepherd | 9 | M | thorax | no | Cutaneous melanocytoma | 3 | 25 | 80 | 2 |
| 70 | Mixed breed | 13 | M | lip | D | Oral melanoma | 2 | 60 | 30 | 2 |
| 71 | German shepherd | 7 | M | lip (skin) | no | Cutaneous melanocytoma | 0 | 10 | 75 | 2 |
| 72 | Yorkshire terrier | 12 | M | penis (skin) | L | Cutaneous melanoma | 6 | 70 | 15 | 2 |

**Supplementary Table 1:** Breed, age, sex, site of the primary lesion, metastases (mets; L=local; D=distant; n/a= not available), histological diagnosis, mitotic count (MC), cellular atypia (ATY), pigmentation (PG), melanophages (MF).

|  | **MARKER EXPRESSION (cells/HPF)** | | | |
| --- | --- | --- | --- | --- |
| **CASE** | **IBA1** | **CD163** | **CD204** | **MAC387** |
| 1 | 69,2 | 58 | 38,4 | 5,6 |
| 2 | 35,6 | 62,4 | 40,8 | 0,0 |
| 3 | 21,2 | 72 | 49,2 | 0,9 |
| 4 | 35 | 59 | 34,8 | 0,6 |
| 5 | 40,2 | 93,6 | 62,8 | 1,0 |
| 6 | 0,4 | 6 | 29,8 | 0,0 |
| 7 | 86 | 113,8 | 60,6 | 7,6 |
| 8 | 67,8 | 64,8 | 87 | 15,6 |
| 9 | 0,6 | n/a | 79,4 | 2,2 |
| 10 | 0,6 | n/a | 41,2 | 0,0 |
| 11 | 39 | 65 | 22,2 | 0,1 |
| 12 | 1,2 | n/a | n/a | 0,0 |
| 13 | 1,2 | 26,4 | 12 | 1,3 |
| 14 | 9 | 83,2 | 82,2 | 2,0 |
| 15 | 0,8 | 0,2 | 0 | 0,0 |
| 16 | 3,4 | 48,2 | 9,6 | 0,0 |
| 17 | 58,6 | 45,6 | 34,4 | 0,4 |
| 18 | 109,6 | 26,8 | 32,8 | 0,0 |
| 19 | 7,4 | 69,2 | 50 | 0,0 |
| 20 | 49 | 119,6 | 39,2 | 0,0 |
| 21 | 66,8 | 94,2 | 68 | 8,9 |
| 22 | 81,8 | 70,4 | 93,4 | 0,0 |
| 23 | 75 | 84,2 | 89,2 | 0,9 |
| 24 | 27 | 35,4 | 73 | 7,3 |
| 25 | 0,4 | 51 | 52,8 | 0,0 |
| 26 | 117 | 116,4 | 139,8 | 8,5 |
| 27 | 86,6 | 122,4 | 53,8 | 31,3 |
| 28 | 52 | 114,2 | 28,2 | 5,2 |
| 29 | 40,4 | 90,8 | 59,4 | 6,3 |
| 30 | 8,3 | 50,6 | 13,8 | 4,0 |
| 31 | 53 | 74,8 | 78,8 | 16,7 |
| 32 | 40,8 | 92,4 | 62,4 | 3,0 |
| 33 | 7,4 | 40,4 | 115,6 | 7,2 |
| 34 | 50,2 | 53 | 21 | 0,1 |
| 35 | 0,6 | 91,6 | 29,4 | 9,9 |
| 36 | 79,2 | n/a | 55,2 | 29,6 |
| 37 | 53,4 | 135,6 | 30,2 | 7,6 |
| 38 | 0,8 | 52,4 | 24,6 | 11,7 |
| 39 | 68,2 | 84,8 | 44,4 | 21,5 |
| 40 | 1 | 16,4 | 73 | 10,7 |
| 41 | 33,2 | 71,6 | 64,8 | 0,2 |
| 42 | 36,8 | 49,6 | 39,6 | 3,4 |
| 43 | 41 | 48 | 27,8 | 3,4 |
| 44 | 23,8 | 52,4 | 31,6 | 5,2 |
| 45 | 58,8 | 71,8 | 52,4 | 28,6 |
| 46 | 58,6 | 29 | 8,2 | 0,8 |
| 47 | 101,2 | 142 | 150,2 | 1,4 |
| 48 | 45,8 | 35,8 | 76,8 | 14,3 |
| 49 | 52 | 92,6 | 49,4 | 28,8 |
| 50 | 44,2 | 38 | 20,6 | 4,3 |
| 51 | 129,8 | 128,8 | 34,4 | 15,8 |
| 52 | 0 | 10,6 | 16,4 | 1,4 |
| 53 | 11,6 | 60,6 | 52,2 | 3,2 |
| 54 | 51,2 | 83,8 | 34,2 | 10,6 |
| 55 | 27 | 66,8 | 40,4 | 0,0 |
| 56 | 0 | 24 | 6 | 0,8 |
| 57 | 3 | 7,2 | 24 | 22,6 |
| 58 | 0,2 | 14,4 | 14 | 0,0 |
| 59 | 1,4 | 57 | 41,4 | 0,0 |
| 60 | 63,6 | 43,2 | 42,4 | 1,8 |
| 61 | 76,4 | 58,8 | 55,6 | 0,0 |
| 62 | 59,4 | 81,8 | 80,6 | 2,2 |
| 63 | 68 | 111,4 | 41,6 | 3,5 |
| 64 | 51,8 | 62 | 38,4 | 7,6 |
| 65 | 53 | 123,2 | 90,8 | 5,9 |
| 66 | 41,6 | 27,8 | 23,6 | 2,8 |
| 67 | 61,2 | 20,4 | 58,2 | 0,0 |
| 68 | 11 | 3,8 | 40,4 | 0,0 |
| 69 | 54,2 | 80,6 | 69,4 | 0,0 |
| 70 | 71,4 | 75,8 | 94 | 0,8 |
| 71 | 61,7 | 44,8 | 32,4 | 0,0 |
| 72 | 44 | 66,8 | 51,4 | 0,2 |

**Supplementary table 2:** Mean number of IBA1, CD163, CD204, and MAC387-positive cells of the 72 cases of the study.
